# Exit interviews administered to patients participating in the COSTOP placebo controlled randomised trial in Uganda

**DOI:** 10.1016/j.conctc.2016.05.008

**Published:** 2016-05-14

**Authors:** Andrew Nunn, Zacchaeus Anywaine, Janet Seeley, Paula Munderi, Jonathan Levin, Ronnie Kasirye, Anatoli Kamali, Andrew Abaasa, Heiner Grosskurth

**Affiliations:** a MRC Clinical Trials Unit at University College London, London, UK; b MRC/UVRI Uganda Research Unit on AIDS, Entebbe, Uganda; c London School of Hygiene and Tropical Medicine, London, UK; d School of Public Health, Faculty of Health Sciences, University of the Witwatersrand, Johannesburg, South Africa

**Keywords:** Cotrimoxazole prophylaxis, Exit interview, Adherence

## Abstract

**Introduction:**

COSTOP was a randomised controlled trial designed to assess the risks and benefits to HIV-infected participants stabilised on anti-retroviral treatment of stopping cotrimoxazole (CTX). In order to assess the extent to which patients may have had access to and used CTX other than that supplied as study drug it was decided to conduct an exit interview.

**Methods:**

A structured interview was administered by interviewers who were not associated with the COSTOP trial team in order to make it easier for participants to respond truthfully to sensitive questions about adherence to the study protocol.

**Results:**

A total of 1993 participants were interviewed. Only 29 (1.7%) said they had taken their left over CTX; 101 (6.1%) had kept supplies at home. When asked about obtaining open label CTX during the trial 92 (4.7%) participants said they had done so, in contrast to only 12 who admitted doing so when asked at trial visits. The questions participants found most difficult to answer honestly at clinic visits were those concerning adherence to trial drugs (15.6% of participants) and whether they had slept under the insecticide treated mosquito nets (14.9%).

**Discussion:**

The exit interview demonstrated that there was some evidence of open label drug being taken by the participants. However, the results from the interview do not suggest that the trial results would have been seriously compromised. We would recommend the exit interview as a valuable way of assessing adherence to trial procedures.

## 1 Background

Cotrimoxazole (CTX) is widely used as prophylaxis against opportunistic infections among HIV-infected persons as recommended by the World Health Organisation [1]. The benefits of continuing to use CTX in patients in limited resource settings who have been stabilised on antiretroviral treatment are unknown. COSTOP was a double blind placebo controlled trial designed to evaluate the benefits and risks associated with stopping CTX in two sites in Uganda [2].

For intervention trials, like COSTOP, that include provider–client interactions, exit interviews with clients are recommended to monitor their understanding of the advice that was provided [3]. Laboratory based tests to detect the treatment under study are often non-existent or expensive, and therefore exit interviews conducted by independent research staff are an important way in which to evaluate adherence to study product [4] Findings from exit interviews have been reported in brief in many accounts of trial findings but the experience of using this technique has only occasionally been presented in standalone papers [5], [6].

Because most patients entering the COSTOP would be expected to have unused supplies of CTX at home and the drug is relatively inexpensive and can readily be obtained over the counter in Uganda the investigators considered it important to assess the extent to which patients might have taken left over supplies or drugs from other sources while participating in the trial. Use of CTX outside of the study clinics could potentially render the results of the trial uninterpretable. A simple test to identify metabolites of CTX in randomly collected urine samples would have been the best way to identify patients allocated to placebo who were taking active drug and to assess whether those allocated to active drug were taking it. Unfortunately no such test is available and although participants were asked at each visit whether they been taking open-label CTX it would have been difficult for them to admit to the study team that they had not followed protocol instructions.

In order to assess the extent to which patients may have had access to and used CTX other than that supplied as study drug it was decided to conduct an exit interview to be administered to all participants by persons not directly associated with the COSTOP trial team in order to make it easier for the participants to respond truthfully to sensitive questions. The present paper reports on the conduct of the exit interview, the findings and the implications for the interpretation of the main trial results.

Only very few clinical trials provide a detailed report on the use and the results of exit interviews to measure adherence in placebo controlled studies. Our paper therefore also provides a contribution to clinical trial methodology.

## 2 Methods

The methods of the main COSTOP study (trial registration number: ISRCTN44723643) have already been reported in detail [2]. In brief, COSTOP was a randomised double blind placebo controlled non-inferiority trial conducted in two clinics in Entebbe and Masaka in Uganda. The study aims were to assess whether stopping CTX is not inferior to continuing with respect to the incidence of pre-defined CTX-preventable events and superior with respect to the reduced incidence of haematological adverse events. Eligible patients were adults aged 18 years or more, infected with HIV who had been receiving antiretroviral treatment (ART) for at least six months, who were clinically asymptomatic having had two CD4 counts (not more than 6 months apart) of ≥250 cells/mm3, the most recent no more than 4 weeks prior to enrolment. Participants were instructed to stop taking their regular supplies of CTX after which they were randomised in equal proportions to receive either CTX provided by the study or a matching placebo; all continued to receive ART as prescribed. Participants were provided with an insecticide treated mosquito net (ITN) and educated about the importance of using it. They were told that both they the study team were blinded to the treatment assignment and that it was important that they did not acquire CTX from any other source. Participants were told to present their COSTOP study appointment cards at the ART service points, private clinics or public health centres and to inform the staff not to prescribe or dispense CTX to them. They were seen monthly for the first three months and every three months thereafter for a minimum follow-up of one year post-randomisation. At each visit patients were seen by a study nurse and trial physician for protocol related assessments. A questionnaire to document adherence to ART and trial drug was administered on each occasion which included a question on whether participants had had access to open label CTX. The trial had 80% power to detect non-inferiority of placebo to CTX with respect to cotrimoxazole preventable events i.e. the upper limit of the one-sided 95% confidence interval of the hazard ratio (HR) for placebo relative to CTX should be no greater than 1.25 (a relative increase of 25%). A total of 2000 participants were required to be enrolled.

The trial was closed after the last randomised patient had completed one year in the study. At the final study visit all patients were requested to participate in an exit interview. They were randomised to be interviewed either by a field worker from the Social Science programme of the MRC/UVRI Uganda Research Unit or by a trained peer interviewer from TASO (The AIDS Support Organisation) in Entebbe or Kampala. A total of eight interviewers (four social scientists and four peers) conducted the interviews using a questionnaire with pre-defined options for answers to most of the questions (see Appendix for details).

## 3 Results

The results of the main study have been presented [7]. In brief they showed that among HIV-infected adults on ART with a CD4 count above 250 cells/mm^3^, discontinuing CTX significantly increased the risk of CTX preventable bacterial infections (particularly pneumonia), and of malaria and led to an increase in hospital admission rates. However, discontinuing CTX also significantly reduced the risk of laboratory determined grade three or four haematological adverse events, in particular neutropenia. The trial found no evidence that discontinuing CTX led to an increase in mortality.

Of 2180 participants enrolled into the trial 37 died before the final visit, 95 were lost to follow-up, 49 had withdrawn consent and six were not interviewed at their final study visit. A total of 1993 remained (1001 allocated to CTX, 992 allocated to placebo) with whom the exit interview was conducted, in 1007 instances by social scientists and 986 by peer interviewers.

The majority of participants, 1667 (83.6%) of 1993, reported that they had supplies of CTX left over at the time they joined the study; 283 (14.2%) said they had none and 43 (2.2%) said they could not recall what they had. Of those with supplies left over at the beginning of the study the majority either submitted them to the study clinic or to their ART provider (Table 1); 101 (6.1%) kept them at home and 29 (1.7%) took them either before or at the same time as taking the study drug. There were minor differences according to the allocated treatment arm and who interviewed the participants.

**Table 1:** Response to question “What did you do with the left over cotrimoxazole at trial entry?”.

	No.	%
Took to study clinic	705	42.3
Took to ART provider	270	16.2
Gave away	275	16.5
Thrown away	213	12.8
Kept at home	101	6.1
Forgotten	70	4.2
Took before or with trial drug	29	1.7
Other	4	0.2
Total with cotrimoxazole left over	1667	100.0

Participants were asked how often they reported having taken the trial drug when they had actually missed some doses; 144 (7.2%) of them admitted to doing so regularly and a further 325 (16.3%) occasionally. In the course of discussions with other participants 430 (21.6%) reported that they were aware that others had found it difficult to admit that they had failed to take the trial drug. There were no differences between those receiving CTX or placebo.

Of 1963 participants who were not switched by clinic study staff to open-label CTX at some time during the trial, 92 (4.7%) admitted to having had access to the drug from other sources, half of them on a frequent basis. There was a slight excess among those prescribed placebo, 50 (5.1%) of 973 compared to 42 (4.2%) of 990 prescribed CTX. The commonest reported source of CTX from outside the study clinic was from HIV treatment providers 48 (53.9%) followed by pharmacies 35 (39.3%). The reasons for obtaining CTX are given in Table 2; the commonest reason being for the treatment or prevention of opportunistic infections. In a small proportion of participants, 12.5% of the 88 who gave a reason, it was on account of having finished tablets provided by the study clinic. These participants were usually travelling and unable to attend for their regular appointment.

**Table 2:** Response to question “Why did you take open label cotrimoxazole?”.

Why taken?	Interviewer				Allocated treatment				Total	
	Peer		Social scientist		CTX		PLC			
	N	%	N	%	N	%	N	%	N	%
OI treatment	16	39.0	14	30.0	15	38.5	15	30.6	30	34.1
OI prophylaxis	10	24.4	19	40.4	10	25.6	19	38.8	29	33.0
Fever	9	22.0	1	2.1	6	15.4	4	8.2	10	11.4
Pain/fatigue	4	9.8	5	10.6	3	7.7	6	12.2	9	10.3
Trial drug finished	4	9.8	7	14.9	7	17.9	4	8.2	11	12.5
Other	1	2.4	1	2.1	1	2.6	1	2.0	2	2.3
Total assessed	41	100.0	47	100.0	39	100.0	49	100.0	88	100.0

OI = opportunistic infection.

No response to question from 4 participants (4 Peer, 1 S Scientist; 3 CTX, 1 PLC).

Three participants (all Peer and CTX) gave more than one reason, 2 pain and fever, one OI treatment and fever.

In these responses, we observed some substantial differences between participants according to their allocated trial arm. For example, the proportion of patients reporting to have taken open label CTX because they had run out of study drug was about twice as high in the CTX group than in the placebo group (17.9% vs. 8.2%), and those who did so to treat an infection or fever was also higher in the CTX arm (53.8% vs. 38.8%). Responses differed also substantially by category of interviewer: for example, among participants interviewed by a peer, about 61.0% stated that they took additional CTX to treat an infection or fever, whilst only 31.9% of those interviewed by a social scientist gave this reason.

Participants were asked which questions they found difficult to answer honestly at clinic visits, the results are shown in Table 3. Being asked about missed trial drugs was the commonest of these questions followed by whether or not they had slept under ITNs. There was a notably higher level of admission to problems when giving an answer to the questions about trial drug and missed ART doses when the social scientists were the interviewers, P < 0.001 for both comparisons. The differences by allocated treatment arm were small.

**Table 3:** Response to question “Which questions did you find difficult to answer honestly?”.

Difficult questions	Interviewer				Allocated treatment				Total	
	Peer		S scientist		CTX		PLC			
	N	%	N	%	N	%	N	%	N	%
Missing trial drug doses	89	9.0	222	22.0	162	16.2	149	15.0	311	15.6
Taking open label septrin	21	2.1	21	2.1	17	1.7	25	2.5	42	2.1
Missing ART doses	27	2.7	74	7.4	55	5.5	46	4.6	101	5.1
Use of insecticide treated nets	153	15.5	143	14.2	140	14.0	156	15.7	296	14.9
All of the above	1	0.1	2	0.2	2	0.2	1	0.1	3	0.2
None of the above	740	75.1	611	60.7	680	68.0	671	67.6	1351	67.8
Total assessed	986	100	1006	100	1000	100	992	100	1992	100

No response from one participant interviewed by social scientist and allocated CTX.

Among other questions addressed to the participants was the frequency of taking the trial drug compared to how often they took CTX before entering the trial (data not tabulated). Responses were evenly split between those saying they took the trial drug more frequently (45.8%) or with the same frequency (43.9%), the remaining 10.3% reported that they took the trial drug less often. There were only small differences by treatment arm, 46.8% of those on CTX and 44.7% of those receiving placebo saying that they took the trial drug more frequently.

A small proportion of participants, 14.3% (13.0% CTX, 15.6% placebo), said they had attempted at some time to guess what treatment they were receiving. Of the 273 who did guess, a significantly higher proportion of those receiving placebo, 99 (65.1%) of 152, guessed correctly compared to 53 (43.8%) of 121 receiving CTX (P < 0.001).

Asked whether a potential trial outcome showing that stopping CTX was safe would mean they would still want to continue to take it, 471 (23.6%) said it was very likely they would want to do so and 46 (2.3%) said it was somewhat likely. However, the majority of patients, 1464 (73.5%) said they would encourage their friends to discontinue taking it if stopping were found to be safe.

## 4 Discussion

The purpose in conducting an exit interview at the conclusion of the COSTOP trial was to attempt to determine the extent to which the outcome of the trial may have been influenced by failure to adhere to the study protocol, resulting in a dilution of differences in the primary outcome between the allocated treatment arms. If more than a small proportion of participants had had access to sources of CTX outside the confines of the study clinics there would have been a serious risk of wrongly declaring the placebo arm to be non-inferior to the CTX arm with respect to the proportion of participants experiencing a CTX preventable event. The exit survey revealed, not unexpectedly, that there were a number of questions related to protocol adherence that participants found difficult to answer honestly at routine study visits. The commonest of these related to missed study drug and use of insecticide treated mosquito nets.

We cannot be sure that the responses given in the exit interview were always truthful. However, the interviews were administered by persons who had had no previous contact with the participants and were not linked to the trial. It is reasonable therefore to assume that participants were more likely to have been honest at these exit interviews; and it is clear from the answers to a number of the questions that participants were prepared to admit to behaviour they had not admitted to trial staff during the trial. In conducting an exit interview of this kind considerable care needs to be attached to the framing of the questions, the training of those administering the questionnaire and assurance given to the study participants that there will be no sanctions imposed for admitting failure to comply with study procedures.

CTX is readily available over the counter in Uganda and in addition most participants would have had supplies at home at the time of entering the COSTOP trial. It is reassuring to note that the proportion of participants admitting to having taken either some of the drug they had left over at home (1.7%) or having obtained drug from other sources during the trial (4.7%) is relatively small although 46 patients (2.3%) admitted to having done so frequently. Of the 92 participants who admitted at exit interview they had had access to CTX during the trial, only 12 had replied in the affirmative when asked during the trial by trial staff about whether they had taken any open label CTX since their previous clinic visit. A reluctance to mention non-adherence while the trial was in progress was corroborated by the findings of a small qualitative sub-study of patient perceptions and adherence to allocated treatment which was conducted in 30 patients (10 who had been allocated to receive active CTX and 20 who had been allocated to matching placebo) during the trial. The findings of this substudy suggested that adherence to study procedures were generally good and there was very little by way of evidence to indicate that patients reported sharing or obtaining drug from other sources. However, while the team conducting this sub-study was not made up of clinic staff, this data collection was conducted while the trial was in progress at the clinic site, so it is possible that there may be some social desirability bias in the responses given. In such a setting it is difficult to know what else could have been done to limit the possibility of participants acquiring CTX outside the study clinics.

It is notable from the exit interview findings that, in the small number of patients who attempted to guess the treatment they had been allocated, a significantly higher proportion correctly guessed they were receiving placebo compared with CTX. There was, however, no evidence to suggest that this seriously influenced their adherence to taking trial drug.

In the comparatively small group of patients who took open label CTX, we observed striking differences between participants from different trial arms regarding the reasons for doing so. Interestingly those in the CTX arm reported more often than those in the placebo arm that they took CTX to treat a perceived infection or fever. Whilst this difference is difficult to understand, it does not suggest that placebo arm participants found it necessary to take CTX more often than those on the active treatment arm. Differences in responses were also observed depending on the kind of interviewer (peer interviewers vs. trained social science interviewers). This is of interest from a methodological point of view. It may well be that these differences were genuine indicating a potential bias depending on the question: more participants responding honestly on some topics to a peer and to others when a social scientist was asking the questions. The potential explanation of the differential responses will necessarily remain speculative.

For many HIV-positive persons whose infection was diagnosed before the availability of ART, CTX was one of the few drugs which provided benefit in the prevention and treatment of opportunistic infections. At the time of planning the COSTOP trial there was uncertainty as to whether those who had been receiving CTX for many years would readily agree to the possibility of stopping it. In addition to providing information on adherence to study procedures the exit interview obtained the views of the participants as to whether they would accept the results of the trial if it showed that stopping CTX was safe. In the event 25.9% indicated that they might wish to continue using CTX and 73.5% said they would encourage their friends to discontinue taking it.

The administration of an exit interview questionnaire to this study population provided valuable information which could not have been readily obtained through trial follow-up visits. It highlighted the fact that there was some evidence of open label drug being taken by the participants. However, taking the results from the exit interview as a whole it seems unlikely that the trial results would have been seriously compromised as a consequence of failure of participants to always adhere to the study protocol.

It is not unusual to ask patients about their opinion of what treatment they have been receiving at the close of a double blind trial although reporting in detail on results from an exit interview is unusual. In one large placebo controlled trial of over 6000 women of a microbicide for the prevention of HIV there was a striking contrast between high rates of self-reported gel use and low proportion of covered sex acts on the basis of an applicator test. However, a sample of 1602 women were interviewed at the end of the trial and many of them admitted they had often failed to use the gel because they forgot to do so or had run out of supplies [8]. This finding was invaluable in understanding the outcome of the trial.

We are aware of very few trials that have published a detailed analysis of exit interview findings as that reported here. On the basis of our findings we would encourage others to consider using an exit interview especially when there is uncertainty about the extent to which participants are complying with the study protocol.

## Conflict of interest

No conflict of interests declared by the authors.

## Authors’ contributions

PM, JL, RK and HG conceived the initial trial idea. AN and JS prepared the initial draft of this manuscript. AA, RK, AK, HG, JL,PM and ZA reviewed and made substantive contributions to subsequent drafts. All authors have read and approved the final manuscript.
