# Supplementary material for: Exit interviews administered to patients participating in the COSTOP placebo controlled randomised trial in Uganda
Source: Contemp Clin Trials Commun. 2016 May 14;3:142–6. doi: 10.1016/j.conctc.2016.05.008 (PMC4918030; doi:10.1016/j.conctc.2016.05.008)
Supplement: Supplementary file 1 [file mmc1.pdf]

Form 26
Participant Exit Interview Form

COSTOP

COSTOP trial number						

Date of form D D | M M M | 2 0 Y Y   Clinic/ hosp. number

Date of birth D D | M M M | Y Y Y Y   Patient initials   Male ❍   Female ❍

Hello. I am _______________________________ from _____________________________

We are working with the MRC/UVRI Uganda Research Unit on AIDS to review your participation in the COSTOP trial which is now at its conclusion. We will be asking you questions about your participation in the COSTOP trial. The information about you and your responses to these questions will be used by the researchers at MRC/UVRI Uganda when reporting on the COSTOP trial, to describe the extent to which participants in either study group of COSTOP complied with the requirements of the study protocol. This information will be very useful in explaining the overall results of the COSTOP trial. All information obtained from this enquiry will be kept confidential and none of it will be presented in such a way that you can be identified.

We are asking your help to ensure that the information we collect is accurate. You may refuse to answer any question or choose to stop the interview at any time.

Do you have any questions about this interview?

Do I have your permission to proceed?

Nkulamusizza Ssebo/nnyabo, amannya gange nze................................... okuva..........................

Tukola n'ekitongole kya MRC/UVRI Uganda Research unit on AIDS okwekeneenya okweetaba kwo mu kunonyereza kwa COSTOP okw'okugezesa okuli mukitundu kyakwo ekyakamalilizo. Tujja ku kubuuza ebibuuzo ebikwata ku kwetaaba kwo mu kunonyereza kwa COSTOP. Amawulire gonna agakukwatako ne by'onooba oyanukudde bijja kukozesebwa abakola ku gw'okunonyereza mu kitongole kya MRC/UVRI Uganda bwebanaaba bawa alipoota ku kunonyereza kwa COSTOP nga banyonyonyola engeri abeetaba mukunonyereza okuva mu bibinja byombi gye bagoberera amateeka g'okunonyereza. Amawulire gano gagya kuba gamugaso okunyonyola ebivudde mu kunonyereza kwa COSTOP okw'okugezesa kwonna awamu. Amawulire gonna aganafunibwa mu kunoonyereza kuno gajja kukuumibwa nga gakyama era tewali ggulire na limu lijja kuweebwayo nga liliko erinnya.

Tukusaba okutuyamba okulaba nga amawulire gonna gwetukunganya matuufu.

Oyinza okugaana okuddamu ekibuuzo kyonna oba okusalawo okukomya okubuuzibwa ebibuuzo akadde konna.

Olina ekibuuzo kyonna?

Nzikirizibwa okugenda mu maaso?

1. What were the main reasons for you deciding to join the COSTOP study? Give as many of the following reasons as you like.
Nsonga/bintu ki ebikulu byewasinziirako okusalawo okwetaba mukunonyereza kwa COSTOP. Mennya zonna ku zino wammanga.

- ❑ Find out whether stopping Septrin is safe
  Okuzuula ng'oba okuva ku septrin tekirina bulabe
- ❑ Try out a new medicine
  Okugezaako edaggala eppya
- ❑ Help others (by participating in research)
  Okuyamba abalala (okwetaba mu kunonyereza)
- ❑ Get treatment
  Okufuna obujanjabi
- ❑ Improve my health
  Okulongoosa eby'obulamu bwange
- ❑ Get CD4 count and other tests
  Okukeberebwa obutafaali bwo'musaayi ne bintu ebirala
- ❑ Financial benefits
  Okufuna obuyambi bwensimbi
- ❑ Other reasons: please specify
  Ne'birala; bimenye
  - o ....
  - o ....
  - o ....

2. At the time of joining the trial what outcome did you expect on your health during the study?
Mu kiseera wewayingirira mu kunonyereza, bibala ki byewali osuubira okufuna mu bulamu bwo?

- ❑ Better
  Birungi nnyo
- ❑ Same
  Tewali njawulo
- ❑ Worse
  Bibi nnyo

3. During the trial you have been asked questions about the following: missing trial drug doses, taking open label Septrin, missing ART drug doses and use of Insecticide Treated Mosquito Net (ITN). Which questions did you find difficult to answer honestly? (Tick all that apply)
Mu kiseera ky'okunonyereza/ky'okugezesebwa, obuziddwa ebibuuzo ku; kuyosa okumira edaggala erigezesebwa, okumira septrin alambiddwa, okwosa okumira eddagala eriweweeza ku kawuka akaleeta siriimu,n'okukozesa akatimba kensiri akalimu eddagala (ITN). Kubibuuzo ebyo, kiriwa ekyakukaluubiriza okuddamu mu butuufu byakyo.

- ❑ Missing trial drug doses
  Okwosa okumira edaggala erigezesebwa
- ❑ Taking open label Septrin
  Okumira septrin alambiddwa
- ❑ Missing ART drug doses
  Okwosa okumira edaggala eriweweeza ku kawuka akaleeta siriimu
- ❑ Use of Insecticide Treated Mosquito Net (ITN)
  Okukozesa akatimba kensiri akalimu eddagala (ITN)
- ❑ All the above
  Byonna ebyo
- ❑ None of the above
  Tewali nakimu kw'ebyo

Interviewer's signature	Print name	Date

Form 26
Participant Exit Interview Form

COSTOP trial number						

Date of form D D / M M M / 2 0 Y Y   Clinic/ hosp. number

4. When you compare your frequency of swallowing Septrin before the trial to swallowing the trial drug during the trial, do you think you swallowed the trial drug more or less compared to Septrin before the trial?
Bwogerageranya emirundi gyewali omira septrin nga tonayingira mu kunonyereza ne'mirundi gyomize eddagala erigezesebwa, olowooza wamira edaggala erigezesebwa emirundi mitono bwogerageranya ne septrin luli ng'okunonyereza tekunabaawo?
- ❑ More
Emirundi mingi
- ❑ Same
Emirundi gyegimu
- ❑ Less
Emirundi mitono

5a. At the start of this study did you have any Septrin left with you from the stock you had before joining the study?
Kuntandikwa y'okunonyereza kuno wali olinawo Septrin yenna eyali asigaddewo ku daggala lyewalina nga tonetaba mukunonyereza?
- ❑ Yes
Yee [nalina]
- ❑ No
Nedda [salina]
- ❑ I don't remember
Sijukira

5b. If yes where did you put it?
Oba yee, waliteeka wa?
- ❑ Threw it away
Nalisuula
- ❑ Brought to COSTOP study clinic
Nalileeta ku clinic ya COSTOP
- ❑ Swallowed it along with the study Septrin
Nalimirira wamu ne Septrin agezesebwa
- ❑ Gave it out to other patients or family member
Namuwa abalwadde/abenganda abalala
- ❑ Gave it to ART service provider
Namugabira abagaba edaggala eriweweeza ku kawuka akaleeta siriimu
- ❑ Other (specify)………………………………………………………………………………
Ekirala [kimenye]………………………………………………………………………………

6. How often did you miss taking the trial drugs but then forget to report it in the interview?
Mirundi emeka gyewerabira okumira edaggala erigezesebwa naye newerabira okukyogera nga bakubuuza ebibuuzo?
- ❑ Never
Tewali [mulundi nagumu]
- ❑ Rarely
Kyali tekitera kubaawo
- ❑ Regularly
Emirundi egisinga

7. How often did you report taking the trial drug during the interview when in fact you had missed some doses?
Mirundi emeka gyewayogera nti wamize edaggala erigezesebwa naye nga mubutuufu wayosezaamu?
- ❑ Never
Tewali
- ❑ Rarely
Nali sitera
- ❑ Regularly
Emirundi egisinga

8a. Did you discuss with other participants in the trial about your experience with the trial drug?
Wanyumyamu ko n'abantu abalala abetabye mukunonyereza ku ngeri gy'osanze mu eddagala erigezesebwa?
- ❑ Yes
Yee
- ❑ No
Nedda

8b. If yes did this encourage or discourage you from taking the trial drug?
Oba yee, kino kyakuwa amaanyi oba kyakumalamu amaanyi okumira edaggala erigezesebwa?
- ❑ Encourage
Kyampa amaanyi
- ❑ Discourage
Kyammalamu amaanyi

9. In your discussions, do you know of any participant in the trial that missed taking the trial drug but found it difficult admitting they had missed when they were asked in the interview?
Nga mukubaganya ebirowoozo, olina omuntu yenna ku betabye mukunonyereza gw'omanyi eyayosa okumira edaggala erigezesebwa naye nekimuzibuwalira okwogera nti teyamira bweyali abuziddwa?
- ❑ Yes
Yee
- ❑ No
Nedda
- ❑ Don't remember
Sijjukira

10a. Did you ever swallow/take Septrin from any other source outside the study for any reason?
Wali omizeeko septrin olw'ensonga yonna ng'omugya mukifo ekirala kyonna ekitali kino?
- ❑ Yes
Yee
- ❑ No
Nedda

10b. If yes, how often did this happen?
Oba yee, kino kyabeerawo emirundi emeka?
- ❑ Rarely
Tekitera
- ❑ Regularly
Emirundi egisinga

Interviewer's signature	Print name	Date

Form 26
Participant Exit Interview Form

COSTOP trial number						

Date of form | D | D | | M | M | M | | 2 | 0 | Y | Y | Clinic/ hosp. number | | | | | | | | | | |

10c. If you took any Septrin from other source, from where did you get it?
Bwoba nga wamira septrin okuva ewalala, wa wennyini we wamugya?

- ❑ Friend/family member
  Mikwano/abenganda
- ❑ TASO or other HIV care organisation
  TASO oba ekitongole ekirala ekirabirira abalina akawuka ka siriimu
- ❑ Bought from pharmacy
  Okugula awatundirwa edaggala

10d. If you took any Septrin from other source, for what reason did you get it ?
Bw'oba wamira septrin okuva ewalala,wamufuna lwa nsonga ki?

- ❑ Prophylaxis of Opportunistic Infections (OIs)
  Kuziyiza endwadde zinakigwanyizi
- ❑ Treatment of Opportunistic Infections
  Okujanjaba endwadde zinakigwanyizi
- ❑ Treating fever
  Kujanjaba musujja
- ❑ Pain/fatigue relief
  Kuweweeza ku bulumi/bukoowu
- ❑ Treating Sexually Transmitted Infections (STIs)
  Kujanjaba ndwadde zakikaba
- ❑ Other (Specify) …………………..
  Ekirala [kimenye]

11. How often did you share your trial drug with other participants or family members?
Mirundi emeka gyewagabana edaggalal lyo erigezesebwa n'abantu abalala oba abenganda zo?

- ❑ Never
  Tewali
- ❑ Rarely
  Sitera
- ❑ Regularly
  Emirundi egisinga

12. If we show that it is safe to discontinue Cotrimoxazole (Septrin) prophylaxis among HIV positive adults stabilized on ART how likely do you think it would be that:
Singa tulaga nti okukomya okumira septrin mu bantu abakulu abalina akawuka nga bateredde bulungi ku daggala eriweweeza akawuka ka siriimu gwe olowooza kiyinza kuba kitya?

12a. If you are stable on ART you would want to regularly continue swallowing Septrin? Would you say:
Bwoba ng'oteredde bulungi ku daggala eriweweeza akawuka ka siriimu, wandyagadde okumira septrin buli lunaku? Wandigambye:

- ❑ Very likely
  Nandiyagadde nnyo
- ❑ Somewhat likely
  Nandyagadde mu
- ❑ Or not likely
  Sandyagadde

12b. Would you encourage or discourage your friends who are stable on ART to discontinue Septrin?
Wandiwagidde/ tewandiwagidde banno abateredde obulungi ku daggala eriweweeza akawuka akaleeta siriimu okuva ku septrin?

- ❑ Encourage
  Nandibawagidde
- ❑ Discourage
  Sandibawagidde

13. Have you ever tried to guess the trial arm you were allocated?
Wali ogeezezaako okuteeba ekibinja ekigezesebwa ky'olimu?

- ❑ Yes
  Yee
- ❑ No
  Nedda

13a. Did your guess influence your adherence to taking the trial drug and/or stay in the study?
Okuteebereza kwo kulina kyekwakola ku ngeri gy'otekeddwa okumiramu edaggala erigezesebwa/oba okusigala mu kunonyereza?

- ❑ Yes
  Yee
- ❑ No
  Nedda

13b. Which trial arm did you presume you were on?
Kibinja ki ekigezesebwa ky'ewasuubira nti mwoli?

- ❑ Septrin arm
  Ekya septrin
- ❑ Placebo arm
  Eky'empeke etariimu kirungo/kigumaaza
- ❑ Don't know
  Simannyi

14. Do you know a safe water vessel (WaterGuard Jerrican orcontainer, etc.)
Omannyi ekitereka amazzi amayonjo? (ekidomola oba kontayina ya WaterGuard, n'ebirala)

- ❑ Yes
  Yee
- ❑ No
  Nedda

15. Do you use a safe water vessel (WaterGuard Jerrican, container, etc.) in your home?
Okozesa ekintu ekitereka amazzi amayonjo (ekidomola oba kontayina ya WaterGuard, nebirala) mu maka go?

- ❑ Yes
  Yee
- ❑ No
  Nedda

Interviewer's signature	Print name	Date
